# Medical termination of pregnancy service delivery in the context of decentralization: social and structural influences

**DOI:** 10.1186/s12939-018-0888-8

**Published:** 2018-11-21

**Authors:** Alana Hulme-Chambers, Samantha Clune, Jane Tomnay

**Affiliations:** 1 Centre for Excellence in Rural Sexual Health, Department of Rural Health, The University of Melbourne, PO Box 386, Wangaratta, VIC 3677 Australia; 2 Department of Rural Health, The University of Melbourne, 49 Graham Street, Shepparton, VIC 3055 Australia

**Keywords:** Medical termination of pregnancy, Decentralization, Service delivery, Rural health, Australia

## Abstract

**Background:**

Medical termination of pregnancy (MToP) is a safe and acceptable abortion option. Depending on country context, MToP can be administered by general practitioners and mid-level healthcare providers in the first and second trimesters of pregnancy. Like other high-income countries, a range of social and structural barriers to MToP service provision exist in Australia. To counter some of these barriers, geographic decentralization of MToP was undertaken in rural Victoria, Australia, through training service providers about MToP to increase service delivery opportunities. The aim of this study was to investigate the factors that enabled and challenged the decentralization process.

**Methods:**

Face-to-face and telephone interviews were undertaken between April and June 2016 with a purposeful sample of six training providers and 13 general practitioners (GP) and nurse training participants. Study participants were asked about their perceptions of motivations, enablers and challenges to MToP provision. A published conceptual framework of synergies between decentralization and service delivery was used to analyse the study findings.

**Results:**

Three key themes emerged from the study findings. First, the effort to decentralize MToP was primarily supported by motivations related to making service access more equitable as well as the willingness of training providers to devolve their informal power, in the form of MToP medical expertise, to training participants. Next, the enablers for MToP decentralization included changes in the regulatory environment relating to decriminalization of abortion and availability of required medication, formation of partnerships to deliver training, provision of MToP clinical resources and local collegial support. Finally, challenges to MToP decentralization were few but significant. These included a lack of a state-wide strategy for service provision, provider concerns about coping with service demand, and provider stigma in the form of perceived negative community or collegial attitudes. These were significant enough to create caution for GPs and nurses considering service provision.

**Conclusions:**

Decentralization concepts offer an innovative way for reframing and tackling issues associated with improving MToP service delivery. There is scope for more research about MToP decentralization in other country contexts. These findings are important for informing future rural MToP service expansion efforts that improve equity in service access.

## Background

Unsafe abortion results in preventable morbidity and mortality; in women of childbearing age 22,800 deaths worldwide are estimated to occur annually [1]. An important reproductive health service, medical termination of pregnancy (MToP) is an acceptable and safe option for women seeking abortion in the first and second trimesters of pregnancy [2, 3]. It involves the use of two medications, mifepristone and misoprostol, and can be safely administered by general practitioners (GPs) and mid-level healthcare providers such as nurses and midwives [4–6]. Administration of MToP in high-income countries by nurse-midwife providers solely has been shown to be effective and acceptable to women [7].

Similar to other high-income countries, abortion laws are inconsistent across Australia, which makes access complex. Although Australia has a federal system of government, abortion law is located in state law [8]. As such, provision and administration of MToP differs widely, existing within state criminal legislation in some Australian jurisdictions [9, 10]. To date, four jurisdictions have decriminalised abortion (although the nature of the legislation differs between these jurisdictions): the Australian Capital Territory, Queensland, Tasmania and Victoria; in the latter decriminalisation occurred in 2008 [8, 9].

Regulations around the availability of MToP in Australia have changed in recent years. The combination of mifepristone and misoprostol for MToP was approved for use in Australia in 2012 (originally this was for pregnancies of up to 49 days but was later revised to 63 days) and were added to the Commonwealth Pharmaceutical Benefits Scheme in 2013 as a subsidised medicine, reducing the cost of the medication [11]. In Victoria, MToP can be commenced in a primary care setting or pharmacy and completed in a home environment [12].

Although these legal and regulatory changes should make MToP more available in parts of Australia, accessibility is hindered by other factors. MToP can only be prescribed by GPs who have undergone mandatory training, within the legal restrictions of each state and territory, and only for use within the first 63 days of pregnancy [8, 9, 13, 14]. There is little information available about which GPs provide MToP [9, 14, 15], but the majority appear to be concentrated in urban locations, creating inequity between rural women and their urban counterparts in relation to provider choice and related costs [12].

Rooted in complex and multilayered belief systems, abortion stigma is the association of negative, socially-ascribed characteristics with women who terminate a pregnancy and/or the medical professionals who provide the service. Abortion stigma is shaped by government, organisational, community and individual level social norms and societal expectations that have and continue to differ over time, as well as within various geographic and cultural spaces. Although the meanings associated with and manifestation of abortion stigma are likely to be context-specific [16], they all act as structural and social barriers to abortion provision and uptake.

Whilst there are relatively few studies that explore abortion stigma, a recent systematic review noted that abortion care providers perceived stigma from colleagues and community about this work [17]. A recent qualitative Australian study involving abortion care providers found that stigma remains an issue for health professionals involved in abortion care [18], and acts as a disincentive for GPs to provide this service [12]. Abortion stigma is also a significant issue for women who have sought or undergone abortion. Studies have noted multiple sources of perceived or enacted stigma including society, community, family, friends, sexual partners, healthcare providers and religious institutions [17, 19, 20]. Such stigma can be harmful to the immediate and long-term wellbeing of women [17, 20, 21].

Decentralization, a concept that spans a range of disciplines including management, social policy, and geography, is variously described with no agreement on a particular definition [22]. As a health system reform mechanism, decentralization is a socio-political process that aims to improve health outcomes by redistributing service delivery from the centre to better address local healthcare needs [23, 24]. Vertical decentralization refers to transference from the centre to the local [25], for example transfer of authority from a central health authority to local health services. Horizontal decentralization is the dispersal of authority across a local context [26], for example a particular service delivered by a range of local health organisations instead of from just one geographic location.

Decentralization may offer opportunities for local communities to be engaged in decisions that affect their health, a means for reducing disparities between rural and urban locations in service access and can potentially improve local level health service coordination [23]. Conversely, decentralization has been criticised for being complex to undertake, prospectively making intended outcomes unattainable and increasing inequity in health service accessibility [27, 28].

Geographic decentralization of MToP services in high-income countries has taken the form of flexible service delivery approaches, such as telemedicine, a technology-based form of service provision which counters distance barriers through consultations via telephone, video or internet [29, 30]. This approach is occurring in a number of countries including Australia and the Netherlands [31]. Other approaches that could be considered as attempts to reduce geographic obstacles to MToP service provision have occurred in countries such as Australia and the United States and have included increasing numbers of GPs trained to provide MToP, reducing provider stigma and addressing professional isolation of providers [32–35].

Most health system decentralization literature critiques health systems on a macro level to understand impacts to health care availability and outcomes. In contrast, there is very little evidence available about decentralization approaches undertaken within a more local context. Further, although in Australia it is problematic that MToP is clustered around urban locations [12], there appear to be no published studies about the geographic, horizontal or vertical approaches to decentralization as a means for reconfiguring abortion service delivery.

The aim of this study was to investigate the factors that enabled and challenged a decentralization effort to increase rural MToP service provision in Victoria, Australia. The approach taken was to increase numbers of MToP providers through delivery of training and professional development sessions to rural GPs and nurses interested in MToP service provision. Using a conceptual framework to analyse the study findings [27], this paper takes a novel approach to investigating the potential for decentralization of MToP services as a means to improve equitable service access for rural women.

## Methods

### Context

Funded through the state government of Victoria, Australia, the Centre for Excellence in Rural Sexual Health (CERSH) has a mission to sustainably develop sexual health service capacity in rural Victoria. In 2013, a conference presentation from the Royal Women’s Hospital (RWH), a major abortion provider located in Melbourne, instigated dialogue about the need for decentralised MToP services, as the Abortion Law Reform Act had been passed in 2008 [35] and MToP had been available since 2012. Subsequently, three key urban-based reproductive health stakeholders (RWH, Fertility Control Clinic, Family Planning Victoria) and a rurally-located organisation (CERSH) collaborated to improve rural pregnancy options and abortion services through training rural GPs and the broader health workforce. Two strategies were developed for delivery in 2014: broader workforce training days provided by all four organisations and professional development evenings for GPs and nurses provided by RWH and CERSH.

Two training days, held in rural Victoria, aimed to increase practitioner knowledge and skills to support rural women experiencing unplanned pregnancy and abortion. These sessions were attended by 83 health professionals, working in various roles. CERSH also organised six professional development evenings for GPs and nurses across rural Victoria, with 97 participants attending. Although in Australia only GPs who have undergone mandatory training are authorised to prescribe MToP, some GPs utilise a nurse-led integrated model of care. In such models the woman’s appointments occur with the nurse and are double-booked with the prescribing GP [11]. The sessions provided information about MToP procedures, including GP training requirements, the role of GP practices in managing MToP service delivery, and establishing local service systems.

### Research methods

This qualitative study was based on individual, semi-structured, in-depth interviews with training providers and training participants. To address the aims of this study, participants’ perceptions about the training and professional development sessions were sought. Purposeful sampling was used to select training participants from which the research team could gain in-depth understandings about the factors that acted as enablers and challenges to decentralization of MToP services. Further, there was limited time and resources available for fieldwork, so purposeful sampling was deemed the best strategy for gaining detailed and informative data in relation to this group of study participants [36].

All six training providers were invited into the study because of their roles in the design and delivery of the training. Their perceptions about the rationale for the training and the effect upon decentralization of MToP services was deemed important to the overall study aim. All providers agreed to an interview. Training providers were female and held senior management and/or clinical specialist positions within key organisations in the sexual and reproductive health sector. Five were located in an urban setting. Training providers were asked about their involvement in establishing the training partnership, previous collaborative work in a rural context, and intended outcomes from the training.

Of the professionals that attended the training sessions, a maximum variation sampling approach was used to invite eighteen health professionals into the study [37]. This approach involved selecting individuals who were known to have been interested in MToP prior to the training, but differed in relation to professional background, age, length of time working in sexual and reproductive health, employment fraction (full or part time), and current work role. All were working in rural Victoria; some by themselves whilst others were in small teams. Thirteen health professionals agreed to an interview and, of these, eleven were female, seven were GPs and six were nurses. Training participants were asked about previous training opportunities in sexual and reproductive health, impact of the training upon professional practice, and challenges and enablers to MToP provision pre and post training sessions. The sample characteristics are presented in Table 1. This study received ethical approval from The University of Melbourne Human Research Ethics Committee (approval number 1646296).

**Table 1:** Sample characteristics

Training providers	Gender	Urban or rural location	Number
Clinical specialist	F	Urban	2
Program executive	F	Urban	3
Program manager	F	Rural	1
Training participants			
General practitioner	M	Rural	2
General practitioner	F	Rural	5
Nurse	F	Rural	6
Total			19

### Data collection

Telephone and face-to-face interviews were undertaken between April and June 2016, with interview length varying between 60 and 90 min. Mode of interview depended on the preference of each interview participant. All participants provided informed consent. For telephone interviews, consent was verbal and captured via audio recording prior to the commencement of the interview whilst written consent was obtained in face-to-face interviews.

### Initial data analysis

Interviews were digitally recorded and later transcribed by a professional transcribing company before being entered into the computer program NVivo (version 10) for data management and analysis. Participants were provided with the opportunity to read and comment upon the content of their interviews prior to the interview being analysed. Two of the researchers (AHC, SC) separately coded each transcript using an inductive analysis approach [37]. This enabled themes to emerge from the transcripts, which later comparison revealed were mostly consensual. Differences in thematic identification were reviewed by JT and further discussion between the research team resulted in refinement of some themes and consensus on the final list of themes.

### Further data analysis

**Fig. 1:**
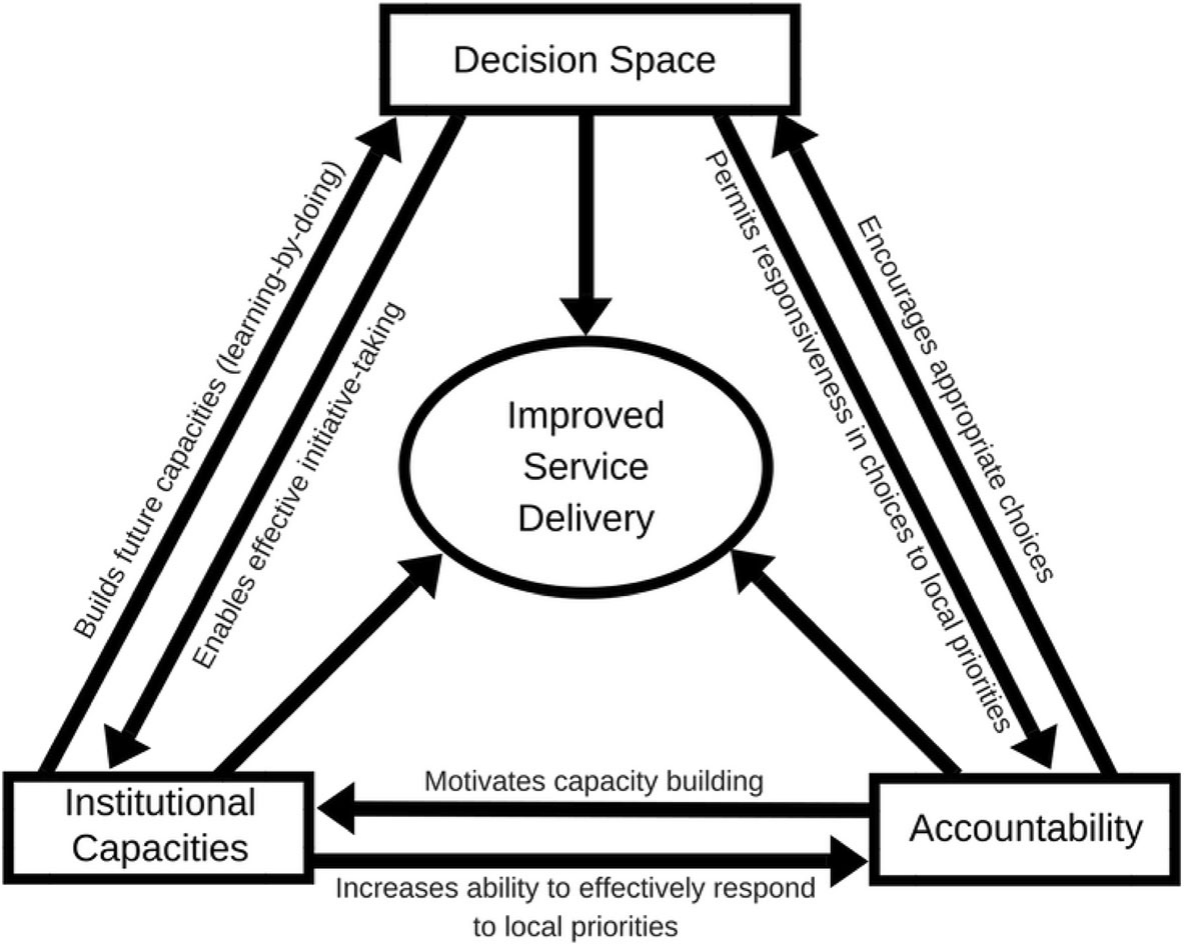
Conceptual framework of synergies between decentralization and service delivery [27]

Having established that themes about the motivations, enablers and challenges to MToP service provision were present in the data, the research team were interested to understand these within the context of decentralization, as this was the premise upon which the training was conceived and instigated and was one of the topics discussed in the interviews. A search of the literature revealed a number of decentralization frameworks [38]. After exploring these, a conceptual framework of synergies between decentralization and service delivery (Fig. 1) was applied as an analytical frame to understand and interpret the study themes [27]. This framework was chosen because of its focus upon different dimensions of decentralization and the impact upon service delivery. The framework authors describe decentralization as about shifting choices of policy and implementation away from central authorities to distal institutions. The framework was developed as a means for empirically analysing the relationships between three dimensions of decentralization for improved service delivery: decision space, institutional capacities and accountability [27]. In this study the framework has been used to understand the decentralization effort undertaken through exploring factors that influenced interactions between decision space, institutional capacities, and accountability, as described by both training providers and training participants.

‘Decision space’ is about how much choice (narrow, moderate, wide) over what functions (e.g., service delivery, access rules, governance) are shifted away from being centrally determined [38]. This space is driven to a great extent by interactions between local decision makers and higher authorities [27].

‘Institutional capacities’ refers to administrative, technical, human and other capacities across multiple levels (system, organisation, individual) [27]. These capacities are important in decentralization implementation [27]. However, the delegating centre and the distal institutions need to have the required capacities to make appropriate decisions within the decision space they occupy [39]. In other words, there needs to be some level of decision making authority held by those involved in relation to the issue under consideration.

‘Accountability’ refers to interactions between local decision makers and elected officials that ensure service delivery is appropriate and responsive to local health needs [27]. Within the context of this paper the term ‘training participants’ has been used instead of ‘local decision makers’ and ‘training providers’ instead of ‘elected officials’ to demonstrate the applicability of the framework to this study, and subsequently explore whether and how accountability manifested between the two groups in the study.

## Results

A total of 19 interviews were conducted. Three key themes were evident in the narratives around decentralization; these being motivations and enablers for, and challenges to decentralization. However, perspectives about these differed somewhat between training providers and participants. Table 2 summarises the themes and the differing perspectives between the two study participant groups. In general, training providers’ perspectives focused on the broader context in which decentralization was being attempted, and the structural factors influencing this process. Training participants spoke more about the factors that influenced decentralization and MToP service delivery at the local level.

**Table 2:** Key emergent themes from the study

Theme	Training provider’s perspectives	Training participant’s perspectives
Motivations for decentralization	Provision of local services for rural women	Provision of local services for rural women
	Reducing demand on urban service providers	
Enablers for decentralization	Urban organisations partnering with a rural organisation	Having access to MToP protocols and resources
	Having a rurally-based organisation provide coordination in the rural setting	Gaining support from rural colleagues also interested in MToP provision
	Changes in the MToP regulatory environment	
Challenges to decentralization	Lack of a clear and defined system for MToP provision throughout Victoria	MToP demand versus supply
		Provider stigma

**Fig. 2:**
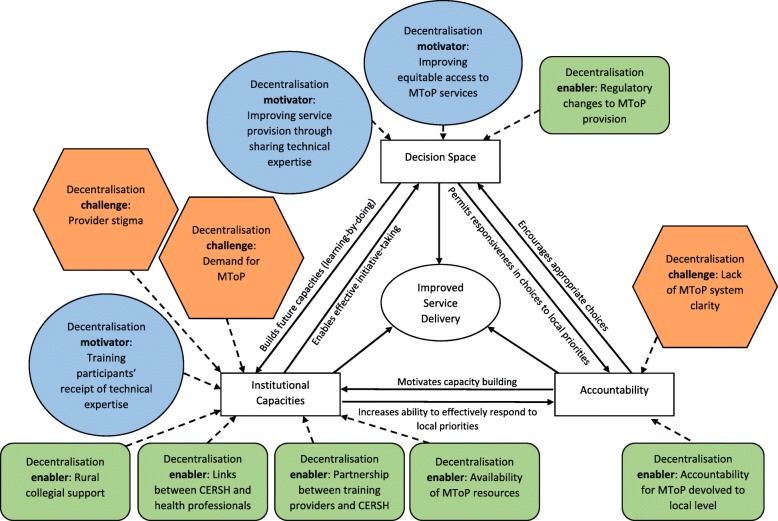
Conceptual framework [27] in the context of the study themes

Figure 2 maps the study themes in relation to where these intersected with the dimensions of the framework.

### Motivations for decentralization

Motivations for and interest in decentralization of MToP were articulated by all participants. Both training providers and participants spoke from a human rights approach, wanting to provide MToP services that were equitable through being rurally based, so reducing time, travel and associated costs for women. Training providers described how, although there had been regulatory change around MToP, services were still largely centralised.

*I just kept thinking about how far women were travelling and I really wanted to get involved with something that went outside of the hospital…something that devolves…I thought well why do you have to do them* [MToP] *in a hospital? You don’t have to…we’ve got the best laws, the most liberal prescribing capabilities…*(GZM, training provider).

Training participants discussed the lack of local abortion service provision in general, as well as the high costs of services that were locally available. They described how clients would be referred to urban services as a result.

*…it was very difficult to get our clients into termination services. We’ve got the one surgical termination service that only operates one day a week and at a significant cost to clients. Our only other option really was sending clients to Melbourne…*(REH, nurse training participant).

*A lot of women were asking about the* [MToPs] *but there was nothing available anywhere, really, outside of Melbourne.* (IEZ, nurse training participant).

GPs and nurses were in favour of the opportunity to decentralise MToP to improve service access.

*I just feel strongly that abortion should be available to people who can’t afford to pay. It just seemed to me to be so logical to push the services away from - we were always sending people to the Royal Women’s, and it’s a pretty big drama to have to go down there for a day, particular if you’re 15 and you don’t want your mum to know.* (PRI, GP training participant).

Training providers’ decentralization narratives similarly focused on the devolution of service delivery from the centre to reduce demand on urban MToP services and improve local service availability.

“…*it* [MToP] *can be done in a local community with trusted health practitioners and appropriate follow up and access to support. It’s cheaper, it’s more available, it can happen more quickly. It allows women to have an abortion earlier in a pregnancy*…” (FID, training provider).

Training participants expressed how having the urban training providers travel to rural and regional Victoria to discuss and support decentralization evoked their curiosity in being able to provide MToP.

*Myself and my colleague both identified that* [MToP] *was something of interest to us….We went with the intention of finding out whether or not this was a service that would be suitable for us to provide…* (HLZ, GP training participant).

*I’ve been a bit aware that it was possible to prescribe but really didn’t know the state of play. So seeing that talk being available – coming up, I thought well I’m going to that to find out what’s going on*. (BOH, GP training participant).

Applying the framework to these findings, it appears that training providers held informal power within the decision space as the holders of medical expertise relating to MToP. In sharing this expertise through training sessions, training providers had a means for shifting mechanisms for service delivery from being centrally delivered to rural locations where health professionals were interested in learning more about MToP. This is reflected in the framework through the arrow ‘builds future capacities’. Training participants were open to change in service delivery flow to the rural context and, within the dimension of institutional capacity, were recipients of technical information that could increase their ability to effectively respond to local MToP service demands.

### Enablers to decentralization

Pragmatic enablers to decentralization were identified and discussed at length by all participants but differed between training providers and participants (see Table 2). Training providers identified three enablers to decentralization. The first was gaining passage into rural Victoria through a rurally-based organisation that was known and trusted by sexual and reproductive health professionals. This was significant because it enabled timely, coordinated reach into geographic areas in which urban training providers had few contacts and little service system knowledge.

*CERSH has got an incredible network…I didn’t really know how well embedded CERSH was in all those communities…they had contacts on the ground…it wasn’t just me going into* [location name] *cold, thinking I wonder who the key gynaecologist is here? They had the local knowledge in those areas* (GZM, training provider).

Partnering with an organisation mandated to work in rural locations was the second decentralization enabler identified by training providers. This was vital for organising training and follow-up service development strategies. This rurally-based organisation acted as the conduit between the urban training providers and the local health professionals.

*She* [worker at CERSH] *had these really strong relationships with people. She was able to pull together a panel of the local people, which we couldn’t have done…We could not have gone in as city workers, I don’t think, into the rural context and provided that.* (AGD, training provider).

The partnership between the urban training providers and the rurally-based organisation created a means for building capacity in rural service providers. Relating to the building of institutional capacity, the partnership was a precursor to providing training as a decentralization approach. The appreciation of CERSH’s local links by training providers indicated the value of a trusted local broker for engaging rural health professionals.

The third enabler discussed by training providers was that the regulatory environment for MToP had changed in Victoria with misoprostol able to be taken in the woman’s home rather than in a hospital setting. This created a tangible means for geographic decentralization.

*…the early medical abortions all had to be admitted to hospital on one day of the week and we were only allowed two beds. To me I thought, well we’ve got to change that. So I guess we decentralised so that we’re now running them in the women’s homes. So the woman does it as an outpatient…*(GZM, training provider).

Training participants identified two key enablers to horizontal decentralization, that is, decentralization within the local context. The first enabler was gaining access to MToP protocols and resources from a rural service that had already commenced MToP service delivery and was willing to share information at the training sessions. Being able to adapt these resources was described by participants as saving time and effort in establishing MToP services.

*Because she* [nurse from organisation that had already established MToP service] *was prepared to share what she had done to set it up, that halved the amount of effort to actually do the next step and say okay I think I can set myself up to do this, because I didn’t have to do - didn’t have to do as much thinking about…the paperwork….*(TOJ, GP training participant).

The availability of MToP resources created an environment conducive to decentralization and service delivery improvement. In Fig. 2 this can be seen via the arrow connecting the institutional capacities dimension to the improved service delivery circle.

The second enabler to horizontal decentralization identified by training participants was gaining support from rural colleagues also interested in MToP. Through attending the training, GPs and nurses saw and talked with local colleagues also interested in MToP service provision. These interactions assisted health professionals to collectively hear and discuss issues relating to rural clinical practice; an opportunity that was strongly valued.

*It* [the training sessions] *also had the added benefit of other people coming, so people coming from all the country towns and from different disciplines. So you got that added benefit of everybody being on the same page.* (RAL, nurse training participant).

The capacities built through access to MToP resources and collegial support contributed to creating a supportive environment in which MToP provision could be considered.

### Challenges to decentralization

Different challenges to decentralization were identified by training providers and participants, reflecting their roles within the MToP environment. Training providers focused on the broader landscape of MToP service provision, identifying the lack of a distinct and visible MToP service system in Victoria as a challenge to decentralization. At the time of the study (2016) there was no state-wide strategy for abortion service provision.

*…so there isn’t a strategy, there isn’t a system of knowing who the providers are, and there isn’t a system of required training, and there isn’t a system of regional access…So there isn’t a system.* (FID, training provider).

*One of the things that is still a limitation to the whole thing is how does a woman find out where there will be someone who would prescribe it in her area? That’s still a big issue*. (GZM, training provider).

Whilst a state government-funded telephone and internet service has since been established that aims to provide health professionals and potential clients with details of MToP providers in Victoria, at the time of the study it was difficult to know which GPs were actively providing MToP services. Relating these findings to the framework (see Fig. 2), a lack of clarity about the broader MToP service system posed challenges to accountability in ensuring local service delivery was appropriate and responsive.

Training participants identified two key challenges to MToP decentralization that reflected their role in direct service provision. First, several training participants expressed concern in potentially not being able to meet demand for MToP once a service was established, and resultant implications for women, including increased waiting times for appointments or having to travel to another area to find a provider.

*I guess we maybe didn’t really consider the impact that would have on us in terms of capacity…We are only one full time nurse five days a week and we have our medical director’s support. So obviously we can only see so many clients. We don’t obviously like to turn people away, so we are very busy. We’ve had to be really careful with how we, I suppose, balance our medical terminations with all our other sexual health things that we do and all the other things that we’re involved in.* (REH, nurse training participant).

Some participants talked about rural GPs, who were MToP providers, already having busy workloads. Further, many of these GPs, who were female, worked part-time and were the only MToP provider in their organisation, creating concerns about who else might be able to provide MToP when the GP was not available.

*…it’s all very well me providing abortion services but I’m not here 100% of the time so how would we cover the scenario when someone just presented at any GP here and said I’m pregnant, what am I going to do?* (PRI, GP training participant).

Second, the potential for provider stigma was articulated as a challenge to decentralization in relation to community or collegial attitudes to service provision. Participants expressed concern in being known as a MToP provider and resultant implications for privacy in rural areas.

*A lot of doctors don’t want to be involved in terminations…even though [town name] is a biggish town, it is small enough that people know who does what, basically. Or it’s easier for people to find out I suppose. So a lot of places, it’s all about the stigma.* (XVT, GP training participant).

*I hope that I don’t get backlash from the town. I’m sure it will raise eyebrows. It’ll be interesting to see. I would hope that my professionalism…in my practice wouldn’t - even if there was some of those people that held anti-abortion views, that they would get past that and still continue to see me as a practitioner, even if they were aware that I was providing that.* (HLZ, GP training participant).

Training participants’ concerns about abortion stigma posed a threat to decentralization through the potential to dissuade these professionals from service provision. This is reflected in the framework (see Fig. 2) as a challenge to the institutional capacities dimension by constraining individual willingness to provide MToP services. The other challenge to the institutional capacities dimension was training participants’ concerns around future ability to meet demand, this also having the potential to impact on increased MToP service delivery.

## Discussion

MToP service provision, despite being an acceptable and safe option that is simple to administer, remains limited in Australia. The aim of this study was to investigate the factors that enabled and challenged the decentralization of MToP in rural Victoria. A conceptual framework of synergies between decentralization and service delivery was used to analyse the study findings [27]. This process revealed that the effort to decentralise MToP services was instigated by motivations to improve MToP access, alongside regulatory change, and this occurred in the decision space. Decentralization is often written about as a political act, driven by policy designed to redistribute functions from central to local government level [39, 40]. What is distinctive about the findings of this study is that the drive to decentralise MToP arose from a desire to improve equity in service availability rather than from a specific policy imperative to do so. In the absence of any policy constraints, and acting within the MToP regulatory environment, the training providers were able to be autonomous in making decisions about training others around MToP, so diluting power around where and by whom service delivery could be undertaken. This circumstance appears to be somewhat exceptional as challenges to the medical control of abortion tend to be controversial [41, 42]. In other country or policy contexts where there is little desire to divest medical power there may also be fewer motivations to decentralise services like abortion. Further, other decentralization efforts may be driven by different motivations other than service access.

Decentralization of health care has been associated with improved equity in service access and has been the focus of a number of decentralization efforts internationally [40]. This study found the events that occurred in the decision space acted as the precursor for subsequent action towards decentralization amongst training providers and participants motivated by a desire to improve equitable access to MToP. Where the confluence of health systems and legal restrictions results in barriers to abortion access [42] social, financial, geographic and personal costs accrued in seeking abortion are exacerbated. Where opportune, decentralization of abortion access not only represents a means to redress these inequities at a pragmatic level but also at a philosophical level, with a recent study finding that abortion is more accepted by populations living in countries with less restrictive abortion policy contexts [43].

Institutional capacities was the site where most of the challenges and enablers to decentralization manifested. This was where practical elements, such as skill development and support, were incubated. However, it was also where challenges to decentralization occurred. Concerns about provider stigma and the potential for overwhelming demand for MToP were barriers to horizontal decentralization because of their occurrence within the local context and their potential to constrain service devolution. Understanding the local context in which decentralization occurs is vital because of the impact upon interdependency between and/or autonomy of health professionals [25]. This is particularly pertinent in rural locations where there are fewer numbers of health professionals, resulting in greater need to work together in partnerships or other collegial arrangements. Further, as found in this study, abortion providers may feel undervalued by society, and experience stress and fear of disclosure in social settings and judgement from colleagues [44]. The ability to undertake actions resulting from vertical decentralization has the potential to be affected by contextual factors relating to professional autonomy, workforce composition and density [27]. Therefore, if an aim of decentralization is to result in health service delivery improvements, decentralization policies need to consider the influence of local contextual factors, particularly in rural areas.

Accountability was the site of least activity in relation to decentralising rural MToP services. This may have been because this study was undertaken 18 months after training sessions had been delivered, and there may been insufficient time for accountability activities to become evident. Further, there is no recognised, state-wide MToP system in Victoria [29] and this could have made it difficult for potential providers to transition from wanting to provide MToP to actually doing so. Clarification around the roles of all stakeholders within a state-wide approach could assist in service planning. This remains an area for further research particularly as the regulatory context surrounding abortion continues to change and evolve in Australia.

Whilst the challenges to decentralization identified in this study were few, they were significant in terms of creating caution amongst some study participants about MToP service delivery, and these remained unresolved throughout and beyond the training sessions. Abortion stigma in particular remains as a broader issue, recognised as a persistent barrier to improving abortion access [41, 42]. Ultimately, it appears that it is not the number of enablers and challenges to decentralization in this case that are significant, but the gravity of each of these in terms of supporting or undermining the dimensions of decentralization. This may be relevant to consider when attempting to improve service availability for other contentious health issues, such as sexual health.

The use of the framework developed by Bossert and Mitchell [27] in this small study may be bold in relation to the application of macro concepts to a comparatively micro decentralization effort. It is significant to remember too that decentralization remains a highly contested concept in relation to how it is defined and whether the effects of a decentralization effort can be attributed to solely to this process [25] so caution needs to be applied to assuming this approach will increase rural MToP service delivery. Regardless, the use of the framework has helped to identify and understand key factors enabling and constraining rural MToP service provision. The study has also contributed to understanding why decentralization attempts may vary in relation to improving health systems, equity in health service availability, and other healthcare outcomes [45].

This study raises important questions to consider in terms of future MToP service delivery developments. First, to what extent could decentralization around a stigmatised health issue, such as MToP, occur equitably in terms of service costs and access, and when there are few incentives for the public system to provide this service? One omission in the framework used is that it does not account for the nature of the program or issue under consideration; a trait common across other decentralization frameworks [38]. It could be useful to contrast a contested health issue to a less politically and socially evocative issue to see if and how a particular health condition influences a decentralization process.

Further, in the context of demand concerns created through establishment of some, but still few, rural MToP services, what are the implications for timely service provision particularly if demand outstrips capacity to provide MToP? Financial incentivisation has been considered as a means to improve MToP service provision [46] but other mechanisms could also be pursued, such as enabling suitably qualified nurses to provide the service independent of GPs, which occurs in some middle and low-income countries [27].

In terms of improved health service delivery through a horizontal decentralization process, patients may be provided better quality of care through cooperation and coordination among healthcare providers [25]. Could a clinical network or community of practice be useful as a means of creating a virtual network of support for rural MToP providers? Further research in this area would be useful to future service system development.

Whilst decentralization holds potential for improving abortion access, it is just one option for scaling up availability of MToP. It is unlikely that MToP will ever be provided by all GPs [47]. In Australia, as well as internationally, there needs to be greater focus on scalability through various direct and supportive options including telemedicine, abortion information hotlines [31], and task-shifting service provision to utilise different models of care, such as nurse-led models [42].

### Strengths and limitations

There are various strengths and limitations associated with this study that need to be taken into account when considering the findings. This study occurred in a high-income country with relatively liberal abortion laws which may mean that findings are not relatable to other country contexts. Training participants were recruited via a purposeful sampling strategy, which may have resulted in researcher bias toward inviting participants supportive of improving MToP availability. Indeed, training participants interviewed indicated an interest in furthering rural MToP service provision, so may have had more positive views about the impact of the training. Such an approach also means that the findings cannot be generalised at a population level. However, the recruitment strategy was helpful in gaining a demographically and geographically diverse sample of training participants from varying backgrounds and levels of experience in sexual and reproductive healthcare provision.

Some interview participants were known professionally to some members of the research team and this may have influenced views about the training. However, participants did also speak at length about the challenges to MToP service provision. Conducting some of the interviews via telephone may have assisted some participants to speak more freely, as telephone interviews can provide a degree of anonymity [48]. The qualitative nature of the interviews meant that the data was rich in meaning and explanations about participants’ perspectives could be explored.

The professional backgrounds of the research team, which are based in nursing, public health, and research expertise in sexual health (and not in the provision of abortion), were a strength because there was a degree of distance between lived professional experiences of the research participants in abortion provision abortion and the professional backgrounds of the research team. This enabled the research team to listen to the narratives without a personal comparative in mind.

The use of a framework for data analysis was a challenge at times as it imposed a level of rigidity in understanding the data. However, at the same time a strength of using the framework was gaining insight into how different threads of discussion regarding service decentralization were linked, enabling contemplation of new perspectives about a complex issue. Finally, whilst qualitative and exploratory, this study contributes to the small body of research that currently exists in relation to improving access to abortion from a health services perspective [29].

## Conclusion

This study investigated the enablers and challenges to a decentralization process which aimed to increase rural MToP service provision. Using a decentralization framework to analyse study findings, it appears that although a number of enablers for decentralization of service provision were generated through the training, the challenges were significant enough to create caution amongst GPs and nurses considering MToP service provision. Whilst MToP remains a complex area of health service provision globally, investigating new ways for improving equitable access remains imperative. This study provides a novel means for reimagining MToP service delivery and suggests further avenues for exploration that may encourage new approaches to improving the availability of this service, particularly in rural locations.

## Abbreviations

CERSH: Centre for Excellence in Rural Sexual Health
GP: General Practitioner
MToP: Medical Termination of Pregnancy
RWH: Royal Women’s Hospital
